# 

**Published:** 1893-06

**Authors:** 


### CONTENTS.

COMMUNICATIONS.

Treatment of the Congenital Deformity of Cleft Palate.................. 261
Landmarks.................................................................. 269
Application for Burns....................................................... 272

PROCEEDINGS.

Mississippi State Dental Association, 1893................................. 273

SELECTIONS.

On the Ethnological Characteristics of the Human Nasal Canals, Considered as an Economic Adaptation.................................. 295
The Nature of Shock and Allied Conditions.................................. 300
The Treatment of Neuralgia of the Trigeminal Nerve.......................... 302
Medical Temperance Association............................................ 304
Notice—Postponement....................................................... 305

NOTICES.

The National Association of Dental Faculties............................... 307
The Annual Meeting of the California Dental Association................... 308
The Missouri State Dental Association...................................... 309
The World's Columbian Dental Congress...................................... 310

EDITORIAL.

The Highways................................................................ 310
Bibliographical.............................................................. 311
Transactions of the American Dental Association............................ 312
Acknowledgement............................................................ 312

